# Application of LightGBM hybrid model based on TPE algorithm optimization in sleep apnea detection

**DOI:** 10.3389/fnins.2024.1324933

**Published:** 2024-02-19

**Authors:** Xin Xiong, Aikun Wang, Jianfeng He, Chunwu Wang, Ruixiang Liu, Zhiran Sun, Jiancong Zhang, Jing Zhang

**Affiliations:** ^1^Faculty of Information Engineering and Automation, Kunming University of Science and Technology, Kunming, Yunnan, China; ^2^College of Physics and Electronic Engineering, Hanshan Normal University, Chaozhou, China; ^3^Department of Clinical Psychology, Second People’s Hospital of Yunnan, Kunming, China

**Keywords:** sleep apnea detection, ECG signal, fusion model, optimisation algorithm, machine learning

## Abstract

**Introduction:**

Sleep apnoea syndrome (SAS) is a serious sleep disorder and early detection of sleep apnoea not only reduces treatment costs but also saves lives. Conventional polysomnography (PSG) is widely regarded as the gold standard diagnostic tool for sleep apnoea. However, this method is expensive, time-consuming and inherently disruptive to sleep. Recent studies have pointed out that ECG analysis is a simple and effective diagnostic method for sleep apnea, which can effectively provide physicians with an aid to diagnosis and reduce patients’ suffering.

**Methods:**

To this end, in this paper proposes a LightGBM hybrid model based on ECG signals for efficient detection of sleep apnea. Firstly, the improved Isolated Forest algorithm is introduced to remove abnormal data and solve the data sample imbalance problem. Secondly, the parameters of LightGBM algorithm are optimised by the improved TPE (Tree-structured Parzen Estimator) algorithm to determine the best parameter configuration of the model. Finally, the fusion model TPE_OptGBM is used to detect sleep apnoea. In the experimental phase, we validated the model based on the sleep apnoea ECG database provided by Phillips-University of Marburg, Germany.

**Results:**

The experimental results show that the model proposed in this paper achieves an accuracy of 95.08%, a precision of 94.80%, a recall of 97.51%, and an F1 value of 96.14%.

**Discussion:**

All of these evaluation indicators are better than the current mainstream models, which is expected to assist the doctor’s diagnostic process and provide a better medical experience for patients.

## 1 Introduction

Sleep apnea syndrome (SAS) is a common breathing-related sleep disorder (Young et al., 2002) characterized by recurrent respiratory arrests during sleep, accompanied by decreased oxygen saturation. This disease is relatively prevalent among the adult population, with a higher incidence rate in males than in females (Young et al., 1993). Globally, it affects approximately 200 million individuals. However, based on statistics related to sleep apnea, about 93% of middle-aged females and 82% of middle-aged males with moderate to severe sleep apnea symptoms have not yet been diagnosed (Young et al., 1997). Sleep apnea is primarily categorized into three distinct types: Obstructive Sleep Apnea (OSAS), which results from dysfunction in the upper airway; Central Sleep Apnea (CSAS), which arises due to neurological abnormalities where the brain fails to generate or convey signals to the respiratory muscles; and Sleep Apnea Hypoventilation Syndrome (SAHS), attributed to diminished air circulation (Gubbi et al., 2012). SAS can occur multiple times during the night and its physiological symptoms include snoring, sleep gasping, waking up with a dry mouth, and poor sleep quality, which can lead to poor concentration, insomnia, cognitive decline, memory loss, and depression (Vanek et al., 2020). Repeated episodes can lead to serious cardiovascular and neurological disorders such as severe coronary syndromes, ischaemic heart failure, cardiovascular dysfunction and stroke (Ancoli-Israel et al., 2003), and they are also associated with daytime fatigue and sleepiness (Vgontzas et al., 2000; Mendonca et al., 2018). As a chronic sleep disorder, SAS is increasingly acknowledged as a significant etiological factor in hypertension and cardiovascular diseases (Caples et al., 2007; Punjabi, 2008). Consequently, precise diagnosis of this condition is imperative.

Polysomnography (PSG) is considered the most accurate way to diagnose sleep apnea (Sateia, 2014), which involves a variety of physiological signals collected from at least 11 channels of different sensors, including respiratory airflow, respiratory movements, blood oxygen saturation (SpO2) electroencephalogram (EEG), electrooculogram (EOG), electromyogram (EMG), electrocardiogram (ECG), etc., in order to determine sleep apnea events (Bsoul et al., 2011). However, due to the large number of wires and sensors connected to the subject, it is cumbersome to wear and requires professional guidance. Although it is a non-invasive technology, it itself will have a negative impact on the subject’s sleep (Byun et al., 2019). In addition, the use of polysomnography is expensive, preventing its use among average families. This limitation is one of the reasons why sleep apnea often goes undiagnosed and undetected in a timely manner (Masa et al., 2013). Therefore, the analysis process requires a significant amount of expert time to evaluate, with an agreement rate of only 80–90% between different experts (Lugo et al., 2020), and there’s a limited number of professionals in medical institutions capable of diagnosing sleep apnea (Hillman et al., 2006; Alghanim et al., 2008; Khandoker et al., 2009). As a result, there is an urgent need to study more convenient sleep apnea detection methods that cater to the patients’ needs.

For this reason, researchers have proposed various SAS detection methods based on different types of single-lead signals, such as respiratory signals (Avcı and Akbaş, 2015), oximetry (Burgos et al., 2010), snoring (Lin et al., 2006), electroencephalography (EEG) (Wang et al., 2020), and electrocardiography (ECG) (Rachim et al., 2014). Among these signals, respiratory and EEG signals are more difficult to collect and have an impact on the sleep itself, while blood oxygen and snoring signals are easy to collect but highly susceptible to interference and have less available information, compared to single-lead ECG signals that are simple to collect and the collection equipment is cheap and suitable for the majority of people to use. In addition, the most important is that the ECG signals can obtain stable information related to the sleep breathing events, which makes it suitable to be used for sleep apnea detection. Therefore, how to effectively detect sleep apnea through ECG signals has become the focus of research.

Currently, machine learning and deep learning algorithms are widely used for sleep apnea detection. As Zarei and Asl (2018) have mentioned: utilized wavelet transform and entropy features in single-lead ECG signals to automatically detect obstructive sleep apnea, which achieves improved classification results. However, the efficiency of this approach decreases with the observation of numerous samples, and it is more sensitive to missing data. Song et al. (2015) proposed an obstructive sleep apnea detection method based on the discriminative Hidden Markov model of ECG signals. However, this approach is highly dependent on each state and tends to perform poorly with long sequence tasks. Hassan (2015) also recorded that utilized the Dual Tree Complex Wavelet Transform for the computerized diagnosis of obstructive sleep apnea using single-lead ECG signals. They compared various models, including Simple Bayes, k-Nearest Neighbors (kNN), Random Forest, Support Vector Machine (SVM), Extreme Learning Machine (ELM), and Regression Analysis (RA). ELM achieved the highest accuracy at 83.77%. Deep learning models exhibit a greater learning capability compared to traditional machine learning models. The main representatives are ElMoaqet et al. (2020) proposed deep recurrent neural network for automatic sleep apnea detection from single-channel respiratory signals; Wang et al. (2019) conducted sleep apnea detection from single-lead ECG signals and utilized an improved LeNet-5 convolutional neural network for automatic feature extraction; Yang et al. (2022) introduced a one-dimensional squeezed and stimulated residual group network, leveraging single-lead ECG signals for obstructive sleep apnea detection; Wang et al. (2022) proposed BI-LSTM, a directed long- and short-term memory network, utilizing single-channel EEG signals for the automatic detection of sleep apnea events. Although the above deep learning-based methods improve the accuracy of OSA detection relative to traditional machine learning techniques, they also have limitations: (1) The majority of research predominantly emphasizes heart rate variability (HRV) while often overlooking vital respiratory parameters associated with SAS, resulting in the suboptimal utilization of the ECG (Feng et al., 2020; Faust et al., 2021). (2) Over-reliance on data results in learning exclusively from existing datasets without assessing the accuracy of the data. High accuracy rates can only be achieved if the data is of good quality. (3) The network architectures utilized in deep learning are relatively complex. With the increase in training iterations, these networks might learn numerous unnecessary features, adversely affecting classification accuracy, elongating training times, and consuming more computational resources.

To address the aforementioned problems, this paper proposes a new method, TPE_OptGBM, which is a LightGBM hybrid model optimized based on the TPE algorithm for sleep apnea detection. LightGBM-based models have been widely used in disease diagnosis (Wang and Wang, 2020; Zhang et al., 2021, 2022). Our proposed hybrid model effectively copes with data anomalies and sample imbalance. First, the abnormal data points were successfully eliminated by introducing the Isolation Forest algorithm to score the ECG data, setting a scoring threshold to isolate the abnormal data. We utilized the algorithm to calculate the sample proportion and balance the samples through undersampling, effectively addressing the data imbalance problem. Due to the numerous parameters inherent in the LightGBM algorithm, traditional methods like random search and grid search are inefficient as they cannot learn from previous optimizations, leading to significant time wastage. In this paper, we adopt a state-of-the-art hyper-parameter optimization framework (Optuna) (Akiba et al., 2019), which continuously learns from previous optimization experiences to automatically adjust the hyper-parameters as needed. This process facilitates obtaining the best hyper-parameter configuration for the LightGBM model. This approach significantly improves the model performance while reducing the time required for parameter tuning. Experimental findings suggest that the integrated fusion model demonstrates superior accuracy in detecting sleep apnea compared to other models and possesses enhanced generalization capabilities.

## 2 Relevant theories and methods

### 2.1 LightGBM model

**Figure 1:**
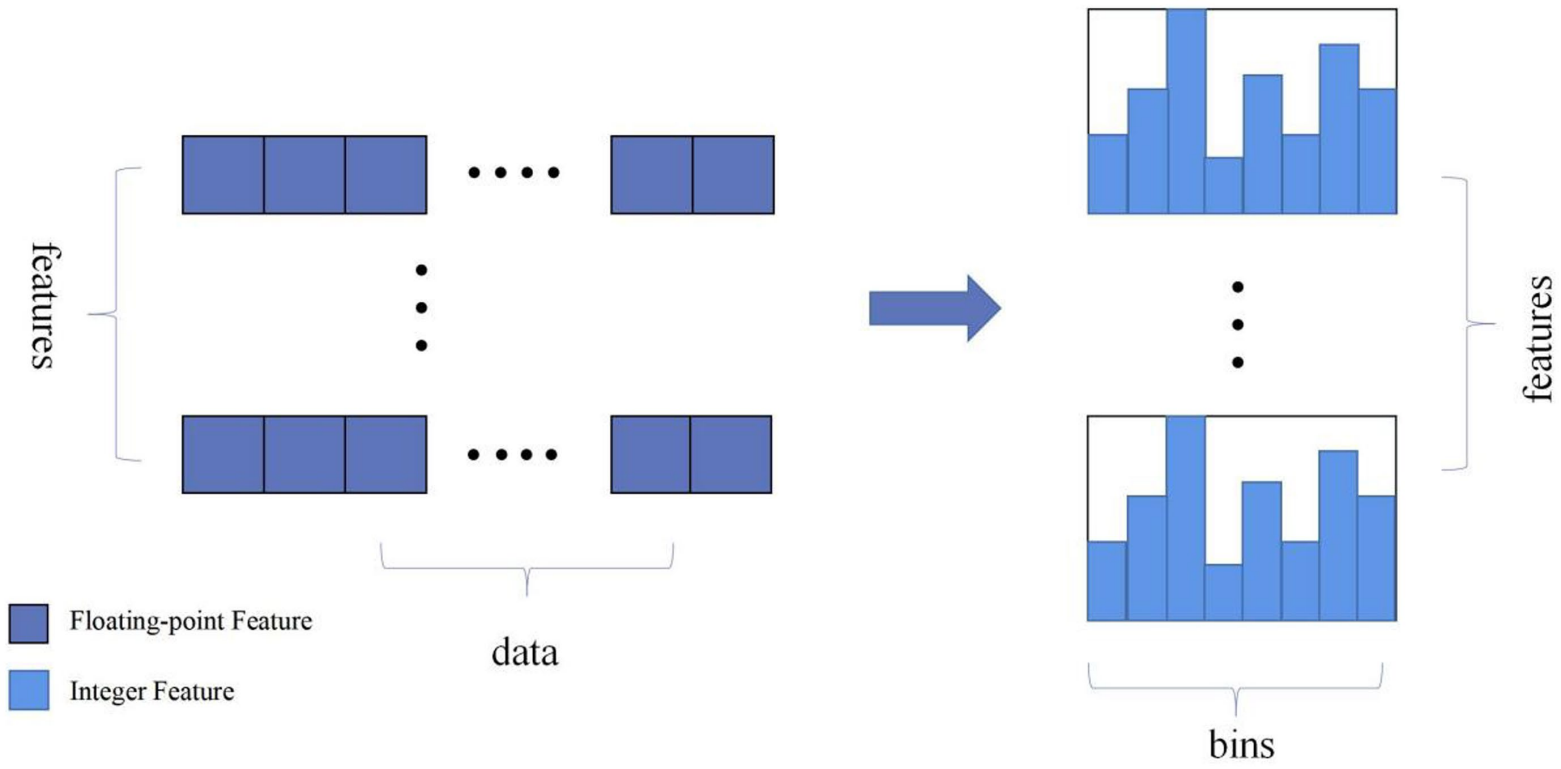
Schematic diagram of the histogram algorithm eigenvalue discretisation. (Discretize continuous floating-point feature values into N integers and construct a histogram with a width of N).

**Figure 2:**
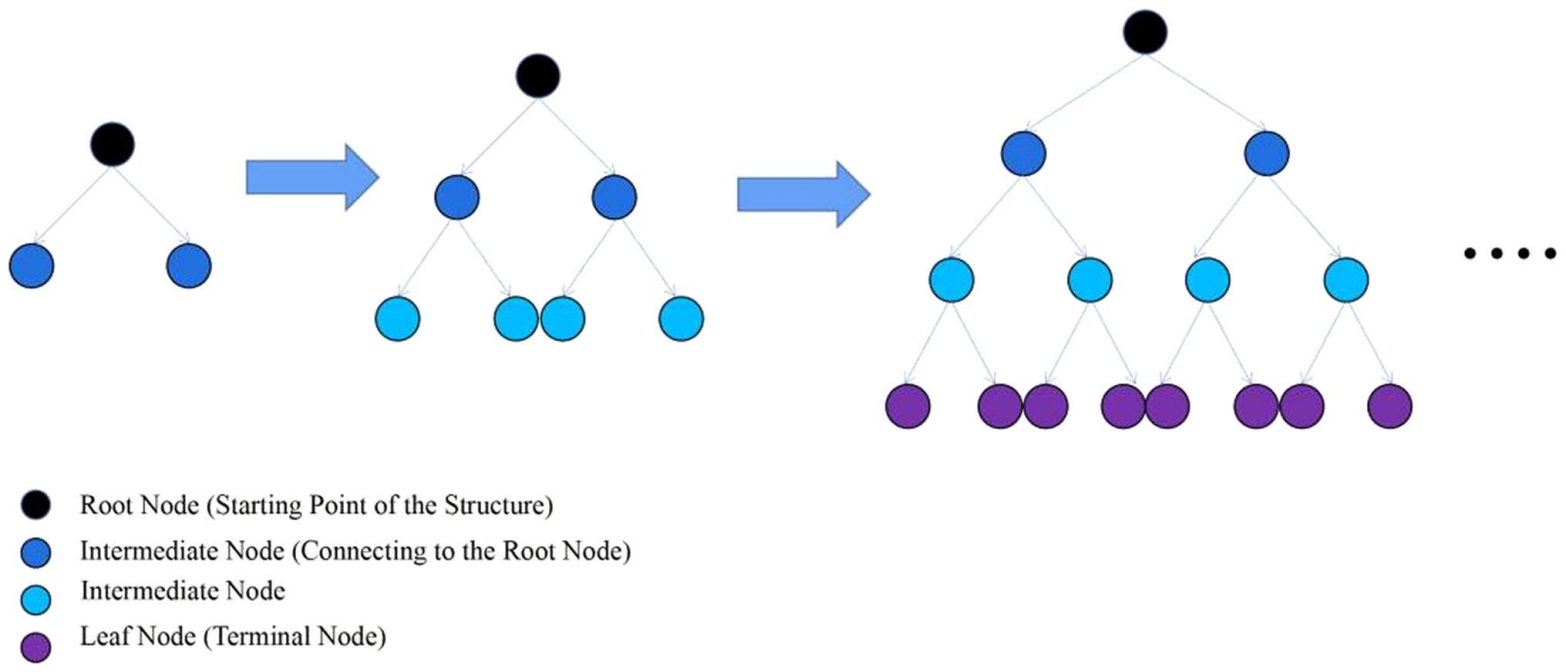
Layer-by-layer growth strategy schematic. (Traversing the data once allows for the simultaneous splitting of all leaves on the same level).

**Figure 3:**
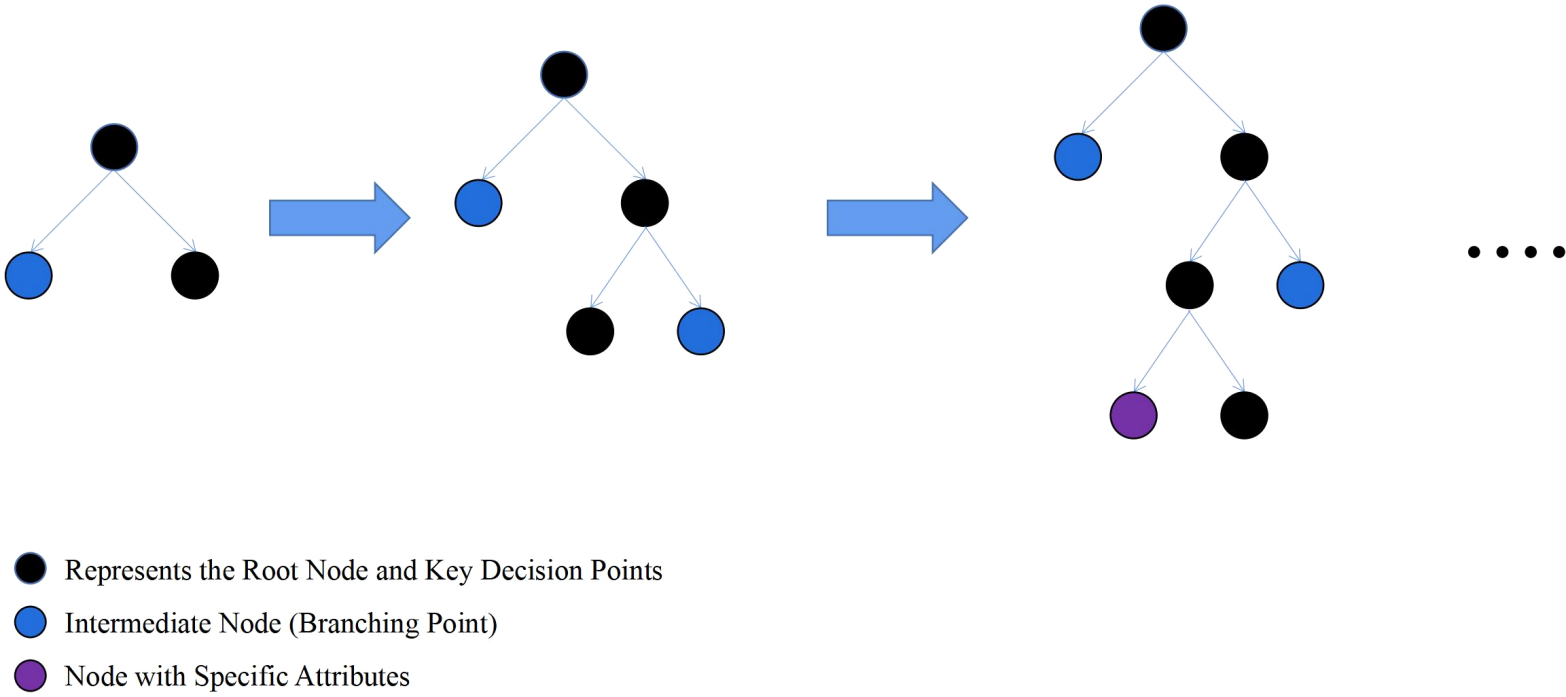
Schematic diagram of leaf-wise strategy growth tree. (Find the leaf with the maximum split gain among all current leaves, and perform the split in the leaf with the maximum gain).

LightGBM is an ensemble learning model based on the improvement of weak classifiers. The main idea of LightGBM model is to use a decision tree to iteratively train to get the optimal model, which has the advantages of good training effect, not prone to overfitting, supports efficient parallel training, and has faster training speed etc. LightGBM mainly adopts Histogram-based decision tree algorithm, which discretizes continuous floating-point features into ‘N’ integer features, which is easy to calculate and store and increases model robustness (Figure 1). The learning algorithms for decision trees mostly generate strategies through a level-wise growth method, which treats leaves on the same level indiscriminately. In reality, many leaves have low split gains, thus leading to a lot of unnecessary computational overhead (Figure 2). LightGBM utilizes a leaf-wise strategy with depth limitation, which can ensure high efficiency while preventing overfitting (Figure 3). Gradient-based One-Side Sampling (GOSS), which retains large gradient samples and randomly samples small gradient samples according to the ratio; Exclusive Feature Bundling (EFB), which reduces the number of features by fusing some features to improving the computational efficiency (Ke et al., 2017).

### 2.2 Objective function for LightGBM model

#### 2.2.1 Objective function

The objective function of LightGBM in the training process contains two parts: one is the loss function, reflecting how well the model fits the training data, and the other is a regularization term that represents the complexity of the model, which is used to prevent the model from overfitting (Equation 1).


(1) $$\begin{array}{c} Obj^{\left(t\right)}=\overset{n}{\underset{k=1}{\sum }}L\left(y_{k},y_{k}^{\left(t\right)}\right)+\overset{t}{\underset{k=1}{\sum }}\Omega \left(f_{k}\right)=\overset{n}{\underset{k=1}{\sum }}L\left(y_{k},y_{k}^{\left(t-1\right)}\right)\\ \;+f_{t}\left(x_{k}\right)+\overset{t}{\underset{k=1}{\sum }}\Omega \left(f_{k}\right) \end{array}$$

This expression means that in each iteration, the model aims to find a new decision tree that minimizes the total loss across all training samples when added to the model, while also considering the model’s complexity to prevent overfitting. Within this context, *t* denotes the total number of iterations; *n* represents the count of all training samples; $y_{k}$ is the actual value for the *k*-th training sample; $y_{k}^{\left(t\right)}$ is the predicted value by the model for the *k*-th training sample at the *t*-th iteration; $f_{t}\left(x_{k}\right)$ is the predictive contribution from the decision tree added during the *t*-th iteration for the *k*-th training sample $x_{k};f_{k}$ corresponds to the *k*-th decision tree model.

#### 2.2.2 Cross-entropy loss function

One of the loss functions that can be chosen in LightGBM, i.e., the cross-entropy loss function, is represented by the whole loss function: For each sample, as long as the model predicts probabilities that are closer to the true labels, the better the model performs. Herein, $y_{k}$ signifies the true label of the *k*-th sample; $e^{-{\hat{y}}_{k}}$ denotes the log-odds of the *k*-th sample being classified as the positive class, representing the raw predictive output of the classifier (Equation 2).


(2) $$L=\underset{K}{\sum }\left[y_{k}\ln\left(1+e^{-y_{k}}\right)+\left(1-y_{k}\right)\ln{\left(1+e\right)}^{y_{k}}\right]$$

#### 2.2.3 Objective function after second order Taylor expansion

To simplify the optimisation of the loss function, a second-order Taylor expansion is used to approximate the loss function, and the objective function obtained is (Equation 3):


(3) $$\begin{array}{c} Obj^{\left(t\right)}=\overset{n}{\underset{k=1}{\sum }}\left[L\left(y_{k},y_{k}^{\left(t-1\right)}\right)+g_{k}f_{t}\left(x_{k}\right)+\frac{1}{2}h_{k}f_{t}^{2}\left(x_{k}\right)\right]\\ \;+\overset{t}{\underset{k=1}{\sum }}\Omega \left(f_{k}\right) \end{array}$$

Within this context, *t* represents the total number of iterations; *n* signifies the total number of training samples; $g_{k}$ is the first-order derivative of the loss function with respect to the model’s prediction for the *k*-th sample, known as the gradient, at ${\hat{y}}_{k}$ ; $h_{k}$ denotes the second-order derivative of the loss function with respect to the model’s prediction for the *k*-th sample, at ${\hat{y}}_{k}$ ; $f_{t}\left(x_{k}\right)$ is the predictive contribution of the new decision tree added in the *t*-th iteration for the *k*-th sample $x_{k}$ . In each iteration, a new decision tree is added to the current model, which is obtained by optimizing the objective function above to minimize the value of this approximated objective function. The newly added decision tree aims not only to minimize the prediction error on the training data (minimizing part $g_{k}f_{t}\left(x_{k}\right)$ and $\frac{1}{2}h_{k}f_{t}^{2}\left(x_{k}\right)$ ), but also controls the complexity of the model, (minimizing part $\Omega \left(f_{k}\right)$ ). The purpose of this is to prevent the model from overfitting.

### 2.3 Isolated forest algorithm

Isolation Forest (IF) is a fast anomaly detection method based on Ensemble. Its core theoretical foundation lies in measuring the degree of isolation between data points by constructing random split trees, called isolation trees, to separate anomalies from normal points, which can be more easily isolated relative to normal points. In a normal dataset, normal points typically require more segmentation steps to be isolated, whereas anomalies can be easily isolated by fewer segments. Isolation trees use a top-down recursive segmentation strategy to split anomalies to separate leaf nodes earlier by randomly splitting the dataset. The main steps can be divided into inputting the dataset and tree-related parameters, outputting the constructed Isolation Forest algorithm, initializing the algorithm, iteratively building Isolation Trees and adding them, and returning the Isolation Forest (Algorithm 1).

**ALGORITHM 1:** 

Pseudo-Code iForest(X,T,H)
**Inputs:** Dataset X,Number of trees T, Maximum height H
Output: Isolation Forest F
1: F = empty forest
2: for i in 1 to t:
3: T = buildTree(X, H)
4: add T to F
5: return F

The main idea of the algorithm is that anomalies are usually more sparsely distributed in the feature space than normal points, and therefore, anomalies are more likely to be isolated. To achieve this, the algorithm randomly selects features and segmentation values in the feature space, making it more likely that anomalies will be isolated to separate leaf nodes at an early stage of segmentation. By generating a set of isolation trees, an anomaly score can be calculated for each data point. The anomaly score is calculated based on the path length of the data point in each tree. Specifically, for each data point, its path length in each tree is calculated and averaged. The shorter the path length, the more likely the data point is to be isolated, and therefore the lower the corresponding anomaly score. The specific process can be divided into inputting the dataset and tree-related parameters, terminating the recursion if the tree has reached its maximum height or the dataset is empty, selecting features and split points, and recursively building subtrees (Algorithm 2).

**ALGORITHM 2:** 

Pseudo-Code function buildTree(X,H) Input: Dataset X, Maximum height H 1: Output: Tree T 2: if H < = 0 or X is empty: 3: create leaf node T 4: return T 5: else: 6: randomly select a feature feat and a split point split 7: partition X into left subset X_left and right subset X_right based on feat and split 8: T = createSplitNode(feat, split) 9: T.leftChild = buildTree(X_left, H-1) 10: T.rightChild = buildTree(X_right, H-1) 11: return T

**Figure 4:**
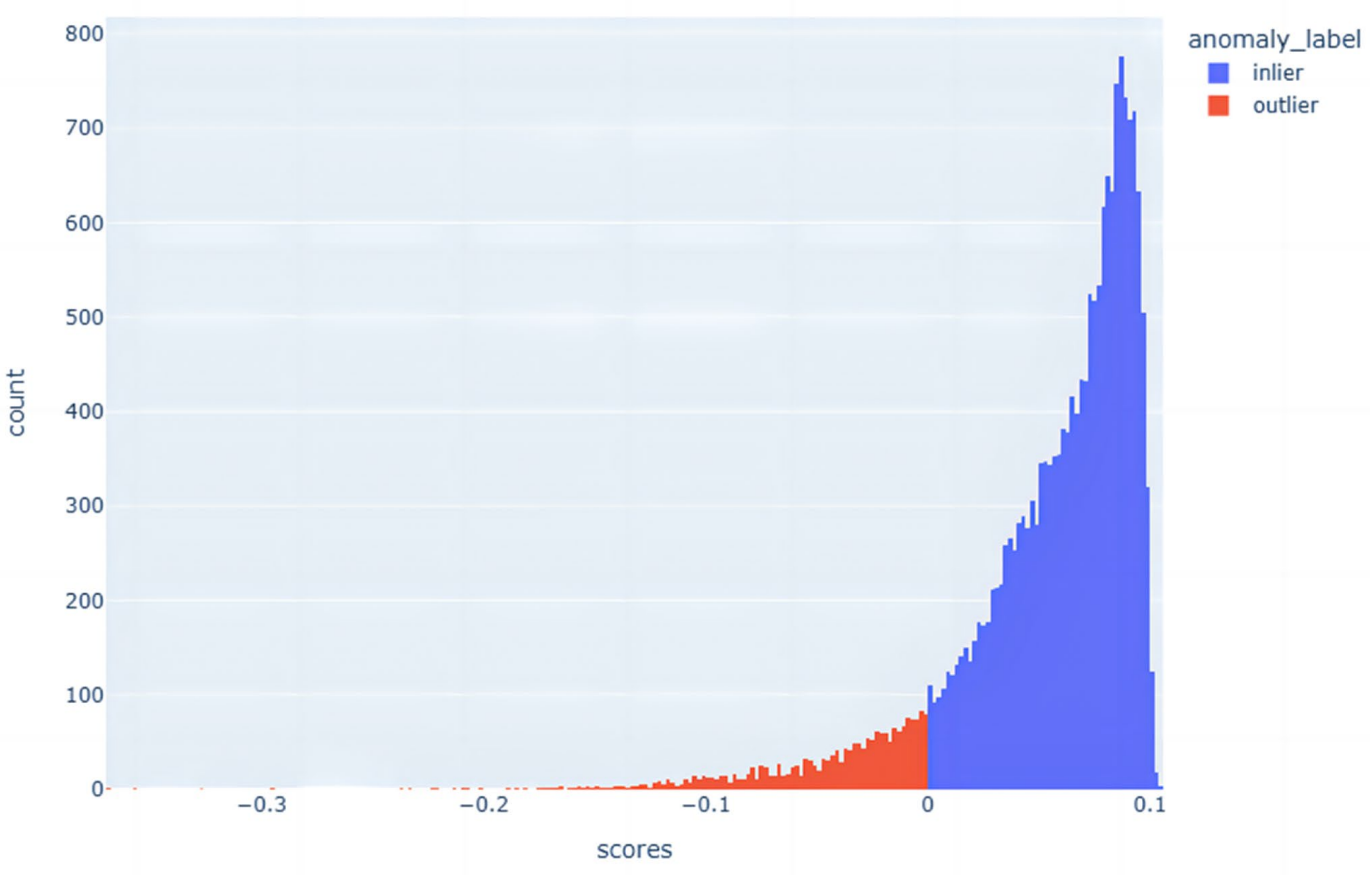
Histogram of the distribution of anomalous and non-anomalous data.

The advantage of the Isolation Forest algorithm is its efficient computational speed and applicability to large-scale data. Due to the construction of random split trees, it is able to quickly detect anomalies in data points and provide low anomaly scores for the anomalies. This method is very effective for dealing with high-dimensional data and large datasets, as it is able to quickly identify potential anomalies in a shorter period of time. The distribution of anomalous data points is shown in Figure 4.

In this paper, the Isolated Forest algorithm is used to measure the degree of isolation of data points in order to identify outliers and non-outliers, and thus effectively detect anomalous data in a dataset. This approach helps to better weed out anomalous data points while balancing the differences between positive and negative samples in the datasets. The stochastic nature of the Isolated Forest algorithm enhances its adaptability to various data scenarios, whether the data involve large-scale, high-dimensional datasets or datasets that are perturbed by noise and outliers. By using multiple isolation trees, the algorithm is able to classify anomalies and normal points more reliably. The advantage of this approach is that it isolates anomalies earlier and reduces their interference with normal samples, thus improving the overall classification accuracy.

### 2.4 Optimisation parameters of Parzen algorithm based on optuna framework

**Figure 5:**
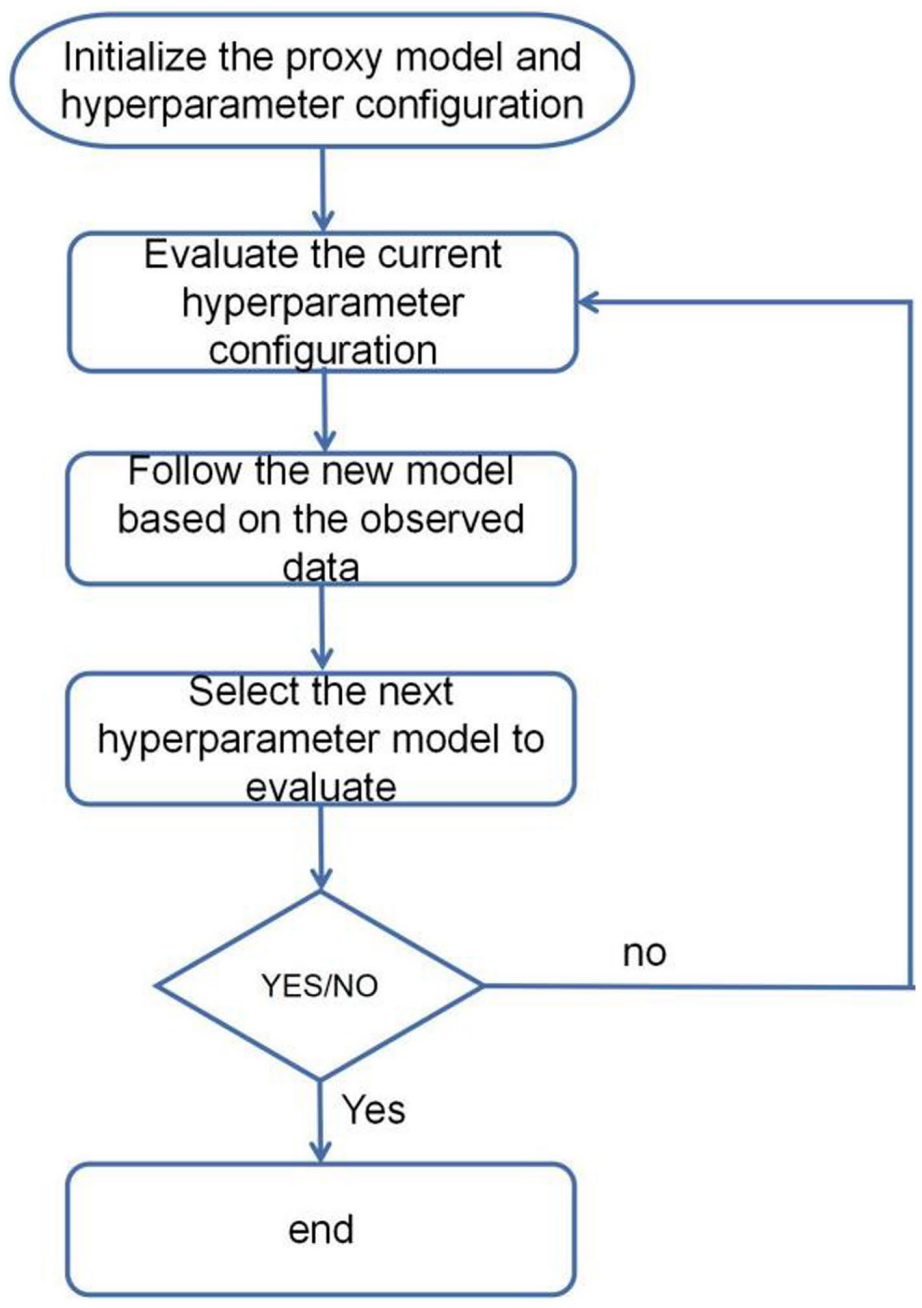
Hyperparameter optimization flowchart.

The fundamental concept of Bayesian hyperparameter optimization based on the Optuna framework involves utilizing Bayesian optimization algorithms to search the hyperparameter space. This process includes evaluating the current hyperparameter configuration, updating the model, selecting the next hyperparameter model for evaluation, and iteratively repeating this cycle to identify the optimal hyperparameter configuration (Figure 5). At its core, this is done by building a proxy model for approximating the potential response surface of the objective function. One of the commonly used proxy models is Gaussian Process Regression (GPR). Gaussian process is a probabilistic model used to describe stochastic processes, in which Gaussian process is used for regression analysis. In Bayesian hyperparameter optimisation, a Gaussian process regression model is used to model the relationship between the hyperparameters and the objective function. This agent model is continuously updated and optimized based on the already evaluated hyperparameter configurations and the corresponding objective function values. By continuously evaluating new hyperparameter configurations and updating the agent model. The Bayesian optimisation algorithm can identify the optimal hyperparameter configuration within a constrained number of iterations. The expression for hyperparameter optimisation (Equation 4):


(4) $$X^{*}=\mathrm{argma}x_{X}f\left(X\right)$$

In this context, X represents a point within the hyperparameter space; $X^{*}$ denotes the optimal solution. Where $f\left(X\right)$ is the optimal hyperparameter configuration for the required solution and $f\left(f\left(X\right)\right)$ is the objective function which receives the hyperparameter $f\left(X\right)$ configuration as input and returns an evaluation metric (e.g., accuracy, loss function, etc.) as the optimisation objective. In this paper, the accuracy rate is used as the evaluation metric for optimisation.

In Optuna, the problem of maximizing the objective function is transformed into a problem of finding the minimizing loss function. Thus, the objective function can be expressed as a negative loss function, where *x* represents a set of parameters. i.e. (Equation 5):


(5) $$g\left(x\right)=-f\left(x\right)$$

In Bayesian Hyperparametric Optimisation, Gaussian Process Regression (GPR) is used as a proxy model to approximate the potential response surface of the objective function $f\left(f\left(X\right)\right)$ . The expression of Gaussian Process Regression can be expressed as (Equations 6–8):


(6) $$f\left(x\right)\approx GP\left(\mu \left(x\right),\sigma^{2}\left(x\right)\right)$$

(7) $$\mu \left(x\right)=K_{*}^{T}{\left(K+\sigma_{n}^{2}I\right)}^{-1}y$$

(8) $$\sigma^{2}\left(x\right)=k\left(x,x\right)-K_{*}^{T}{\left(K+\sigma_{n}^{2}I\right)}^{-1}K_{*}$$

In this context, GP denotes a Gaussian Process, a probabilistic model employed for regression tasks; $\mu \left(x\right)$ is the mean function of the Gaussian Process, representing the predicted value of the objective function; $\sigma^{2}\left(x\right)$ is the variance function of the Gaussian Process, indicating the uncertainty in the prediction of the objective function; $K_{*}^{T}$ represents the covariance vector between the new input *x* and the training inputs; $\sigma_{n}^{2}$ is the noise term added to the diagonal of the covariance matrix to ensure its invertibility; I signifies the identity matrix; y is the vector of observed values of the objective function for the training inputs; $k\left(x,x\right)$ is the covariance of the new input *x* with itself. In each iteration, it updates the parameters of the agent model by using the observed data of the already evaluated hyperparameter configurations and objective function values. The posterior distribution can be obtained by Bayesian inference of the observed data., $\hat{p}\left(x\right)=\frac{1}{n}\overset{n}{\underset{i=1}{\sum }}\delta \left(x-x_{i}\right)$ , $D=\left\{x_{1}x_{2}x_{3}.\ldots x_{n}\right\}$ indicates observed data. Based on the posterior distribution, the next configuration of hyperparameters to be evaluated $x_{\mathrm{next}}=\mathrm{argmaxEI}\left(x\right)$ can be chosen to maximize the desired improvement in the objective function. $EI\left(x\right)=E[\max\left(f_{\text{min}}-f\left(x\right),0\right]$ is the expected improvement function. It usually use certain sampling strategies (e.g., pruning, adding noise, etc.) to balance the use of observed data and the exploration of unobserved data in order to better optimize the objective function and progressively converge to a better hyperparameter configuration.

By iteratively updating the agent model and selecting the optimal sample points and hyperparameter configurations, Bayesian hyperparameter optimisation is able to find better hyperparameter configurations within a limited number of iterations, thereby improving the model performance and efficiency. The agent model can use historical observation data to guide the search process, making the search more intelligent and avoiding the blindness of traditional grid search or random search methods. It is able to make full use of the information of the evaluated sample points and objective function values to intelligently explore and utilize the hyperparameter space to find the optimal hyperparameter configuration. The TPE_OptGBM model proposed in this paper uses algorithms from the Bayesian Optimisation Library. In this paper, the TPE algorithm is improved by abandoning the traditional algorithm of reducing the loss downwards to improve the accuracy, and instead optimizing upwards with accuracy as the optimisation metric.

### 2.5 TPE_OptGBM fusion model

**Figure 6:**
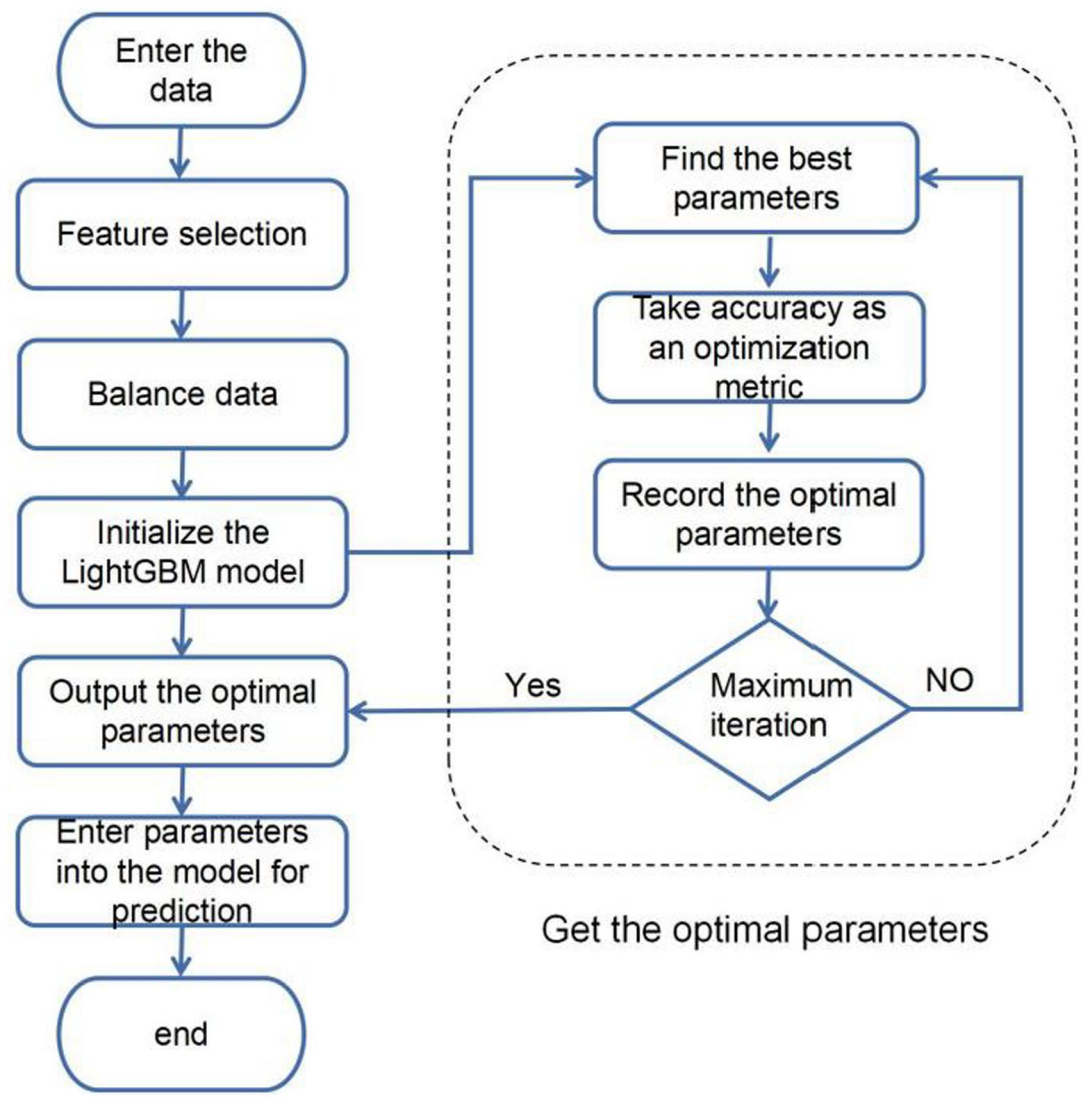
Fusion model flowchart.

In order to improve the accuracy of sleep apnea detection based on ECG signals, a systematic approach was implemented in this study. Initially, the Isolated Forest algorithm is introduced for the screening of abnormal data points, addressing outliers effectively. Given the significant imbalance in the data, with diseased samples being notably fewer than normal samples, an undersampling strategy is employed to balance the differences between positive and negative samples in the dataset. Subsequently, an enhanced Bayesian hyperparameter optimization algorithm is utilized to automate the search for the model’s optimal parameter configuration. Ultimately, the identified optimal parameter configurations are input into the hybrid model for data training. The overall structure is illustrated in Figure 6.

## 3 Data sets and pre-processing

### 3.1 Data sets

The sleep apnea-ECG dataset used in this study come from the [Apnea-ECG Database v1.0.0 (physionet.org)] (Akiba et al., 2019; Goldberger et al., 2003), and consisted of 70 single-lead ECG signals, divided into a training set containing 35 records (a01 to a20, b01 to b05 and c01 to c10) and a test set of 35 records (x01 to x35). Each record contained a continuous digitized ECG signal from the patient for a full night, with recording times ranging from 401 to 578 min. The subjects contained males (57) and females (13), with ages ranging from 27 to 63, heights ranging from 158 to 184 cm, and weights ranging from 53 to 135 kg (Table 1).

**Table 1:** Dataset description.

	Length (minute)	Non-Apnea (minute)	Apne (minute)	AI	HI	AHI	Age	Height (cm)	Weight (kg)
Min	401	11	0	0	0	0	27	158	53
Max	578	535	534	86.8	57.1	93.5	63	184	135
Avg	481.82	305.17	305	21.83	12.38	28.04	45.14	175.84	86.75
SD	±31.57	±156.57	±172.27	±24.10	±9.42	±27.57	±10.83	±5.58	±20.73

### 3.2 Data pre-processing

Firstly, a consecutive full night of ECG signals was segmented, with each segment having a length of 60 s, from which a total of 34,313 segments of data was obtained. In processing the ECG signals for each minute, the following steps were perfsormed:

Signal sampling and filtering: The sampling rate of the signal is adjusted to 100 Hz. The ECG signal was filtered using a Chebyshev band-pass filter to remove noise and baseline drift from it.

R-R interval analysis: The time interval between two adjacent R waves (ventricular contractions) of the heart is extracted from the ECG signal and is called the R-R interval. This interval is used to study heart rate variability, i.e., changes in heart rate over time (Wang, 2019).

Analysis of R-wave amplitude variations: The R-wave Amplitude Modulation Periodicities (RAMP) method was used to analyze the pattern of change of R-wave amplitude and period in the electrocardiogram (Varon et al., 2015).

ECG-derived respiratory signal extraction: generates a respiratory signal in the ECG called electrocardiographically derived respiratory signal (EDR). This signal is obtained by measuring the resistance (R) and capacitance (C) in the ECG and provides information about the heart function (Singh et al., 2020).

**Figure 7:**
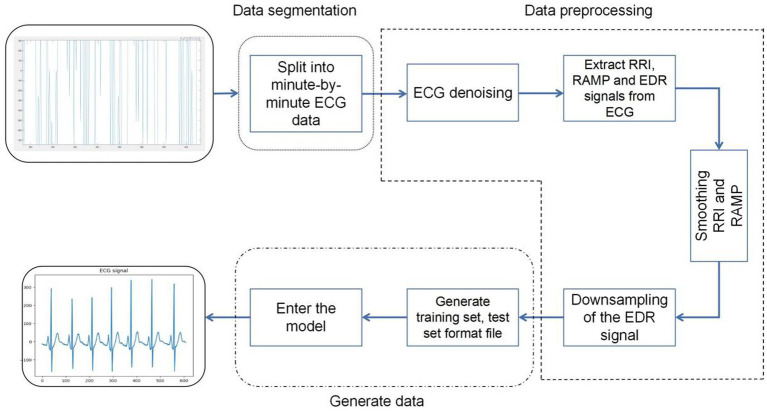
Data preprocessing flowchart.

Signal processing and formatting: The extracted signals are subjected to spline interpolation and smoothing to fill in the missing data, and then the signals are down-sampled to retain the key information while reducing the amount of data. Ultimately, the processed electrocardiogram (ECG) signals were transformed into structured training and testing datasets, facilitating the analysis and evaluation of the model. Special attention was paid during this transformation process to minimize potential information loss during data conversion. Consequently, the ECG signals were meticulously formatted into two-dimensional vectors. This format not only preserves the integrity of the data but also aligns well with the processing requirements of the model. By providing direct numerical information, these vectors enable the model to capture traits more effectively and analyze key features of the ECG signals, thereby enhancing the model’s analytical capabilities and predictive accuracy (Figure 7).

## 4 Experimental results

### 4.1 Experimental environment

Experimental environment: the experiment was run on a computer with Windows 10 operating system. The CPU is Intel(R) Core(TM) i7-10700F CPU @ 2.90GHz 2.90 GHz. The GPU is NVIDIA GTX3060 and the space size is 12GB. The computer running memory size is 64GB. The experiment is run on the Python 3.6 and tensorflow environment.

### 4.2 Assessment of indicators

In this paper, accuracy, precision, recall rate, F1 score, ROC curve and P-R curve are used as evaluation indicators to evaluate the classification performance of the model.

The accuracy rate is the proportion of the number of samples that were correctly predicted to the total number of samples. The formula for calculating the accuracy rate is as follows (Equation 9):


(9) $$Acc=\frac{TN+TP}{TN+TP+FN+FP}$$

Precision is the probability that the sample is actually positive out of all the samples that are predicted to be positive. The precision is calculated as follows (Equation 10):


(10) $$Pre=\frac{TP}{TP+FP}$$

Recall is the probability that it is correctly predicted in all positive samples and is calculated as follows (Equation 11):


(11) $$Rec=\frac{TP}{TP+FN}$$

When the sample ratio is unbalanced, there is often a bias in evaluating model performance by precision and recall. Good results for one metric and poor results for the other, the model’s capability cannot be accurately assessed. For this reason, we introduce the F1-score by weighted average recall and precision values. The F1-score is higher only if the recall and precision results are good. The formula for calculating the F1-score is as follows (Equation 12):


(12) $$F1=2\times \frac{Pre\times Rec}{Pre+Rec}$$

The ROC curve is a curve plotted on the axes of (FPR) and (TPR), which is a measure of the classification problem, and the closer the ROC curve is to the upper left corner, the better the model performance. Recall is used as the horizontal axis and precision is used as the vertical axis. AP is the area of the graph enclosed by the PR curve and the *x*-axis, and the model performs best when the AP value is 1.

### 4.3 Optimisation results

**Figure 8:**
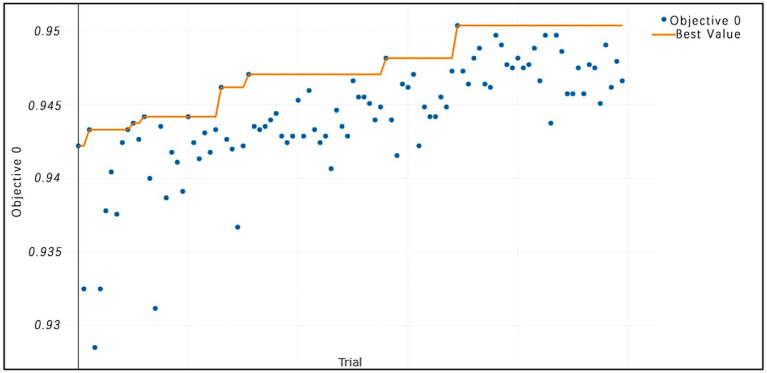
TPE_OptGBM model iteration accuracy. (The horizontal axis represents the sequence of trials, the vertical axis represents the value of the objective function, the blue dots indicate the objective function value for each trial, and the orange line shows the best objective function value obtained so far).

**Figure 9:**
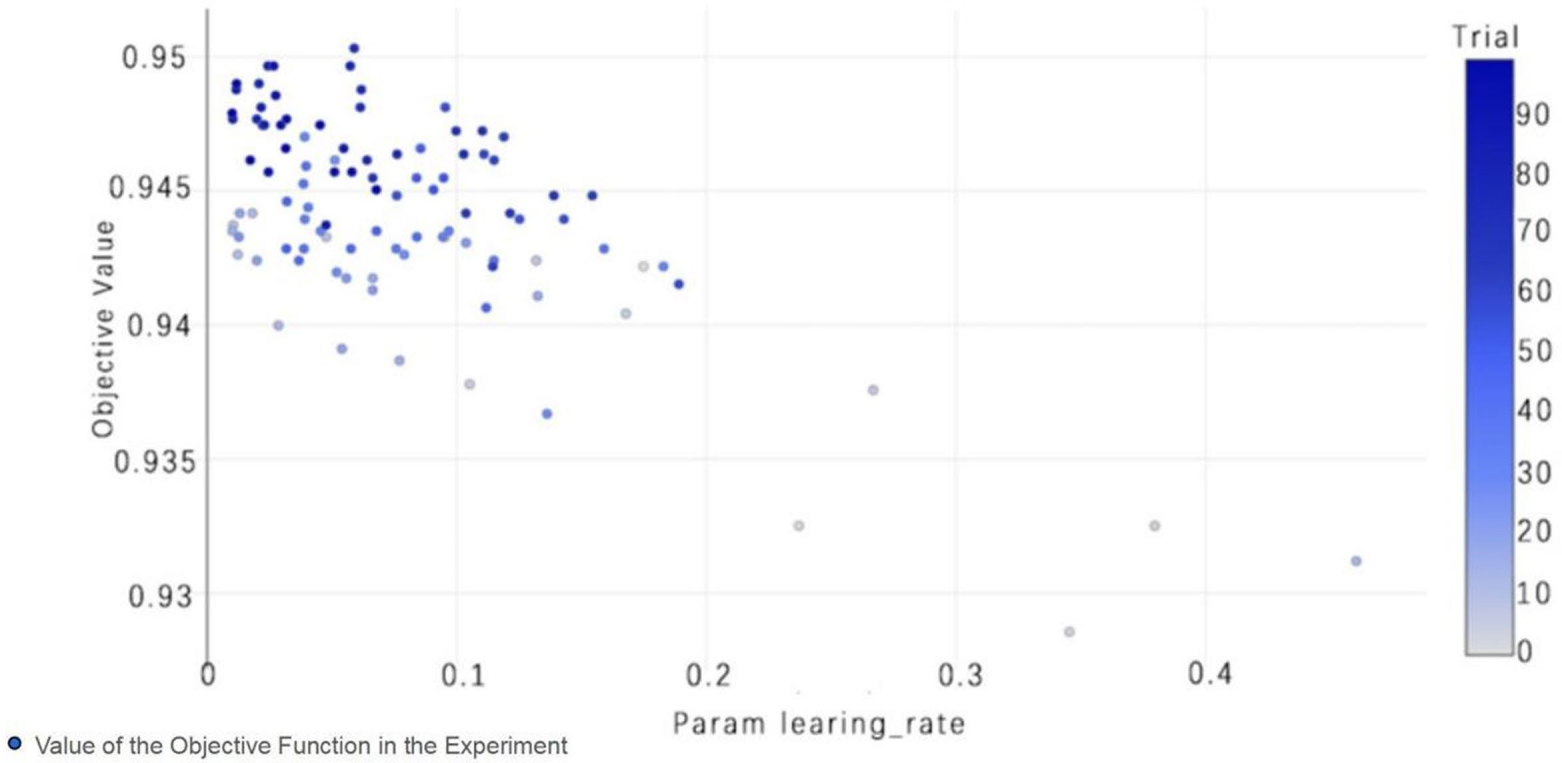
TPE_OptGBM model param learning_rate. (The chart shows the impact of the learning rate on accuracy; as the learning rate parameter changes, the objective function value also changes, thereby selecting the optimal learning rate to optimize model performance).

**Figure 10:**
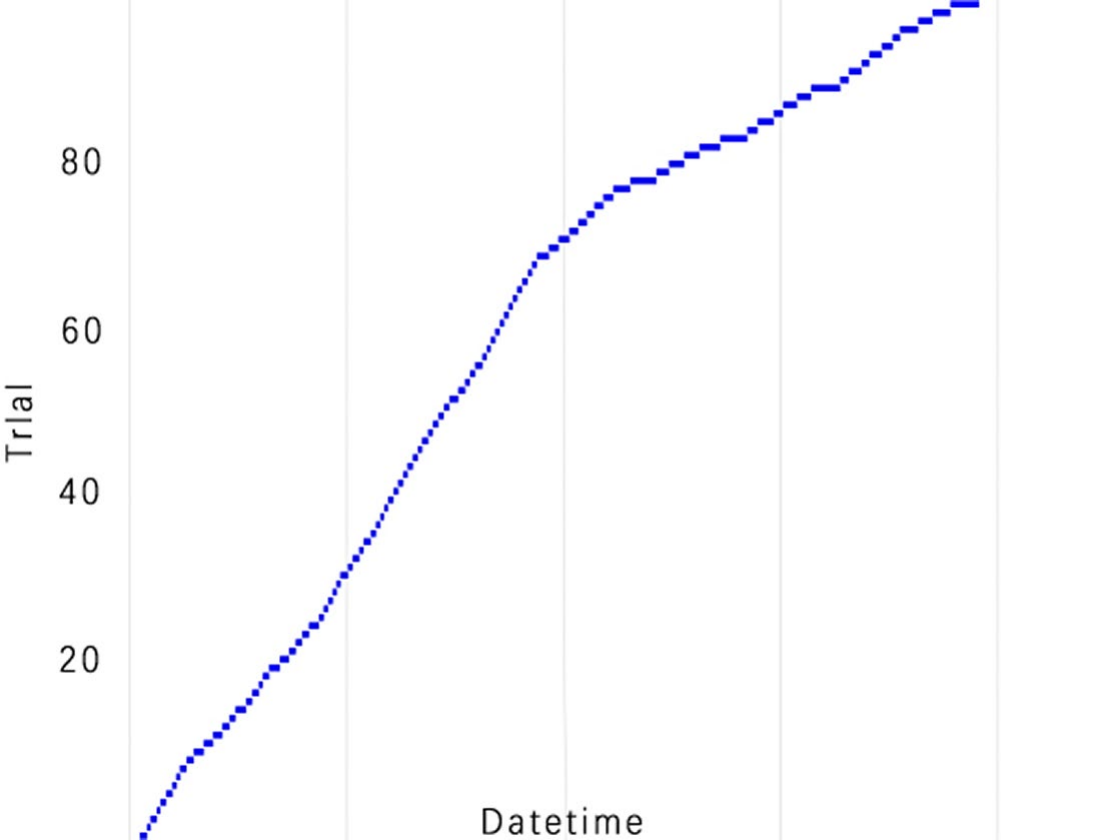
TPE_OptGBM model experimental efficiency. (This is a chart of cumulative trial numbers over time, with the horizontal axis representing time and the vertical axis representing the number of trials, showing that the number of trials increases more quickly at certain points in time).

**Figure 11:**
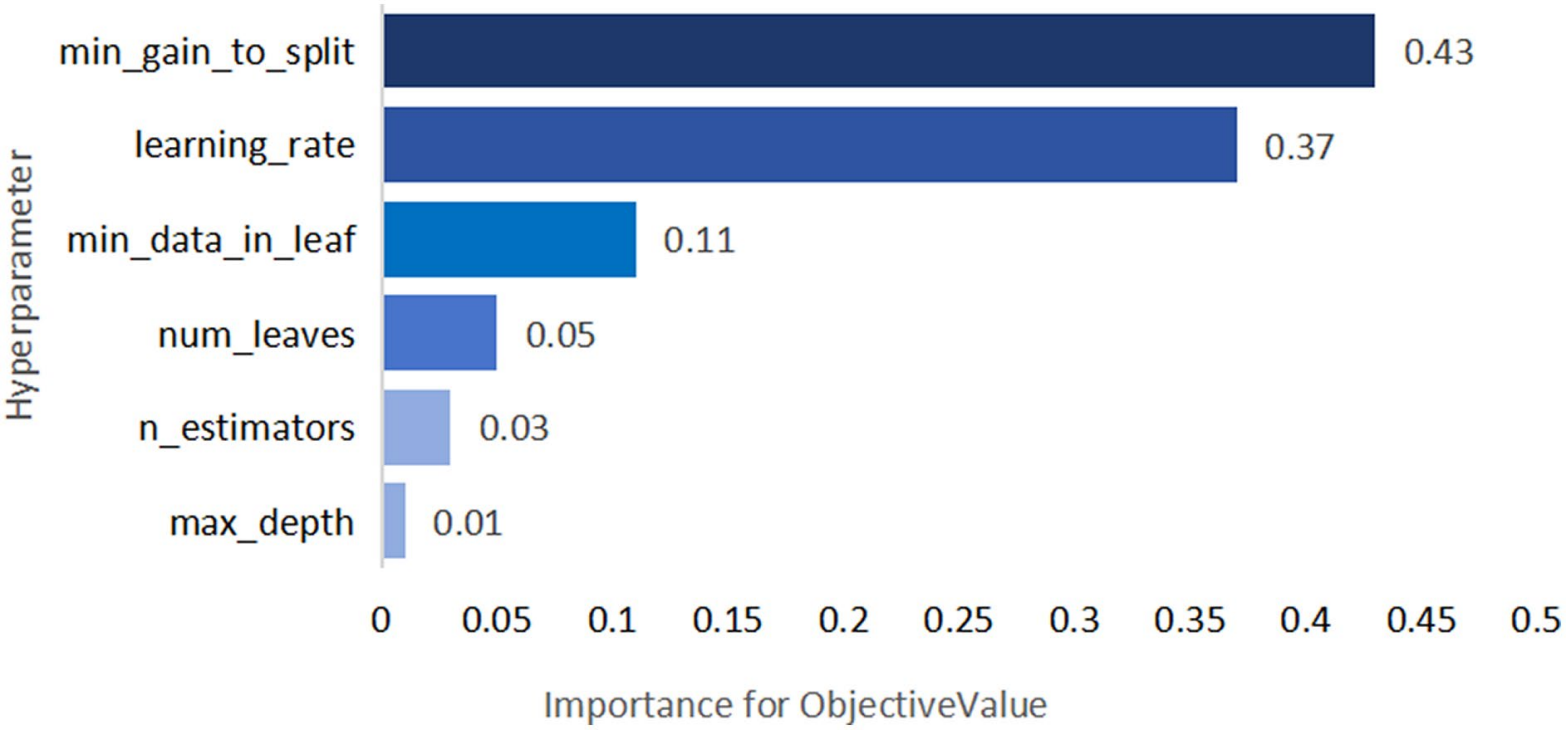
TPE_OptGBM model hyperparameter importance. (Describe the degree of influence of different hyperparameters on the model's objective function value; the larger the value, the more significant the impact).

**Figure 12:**
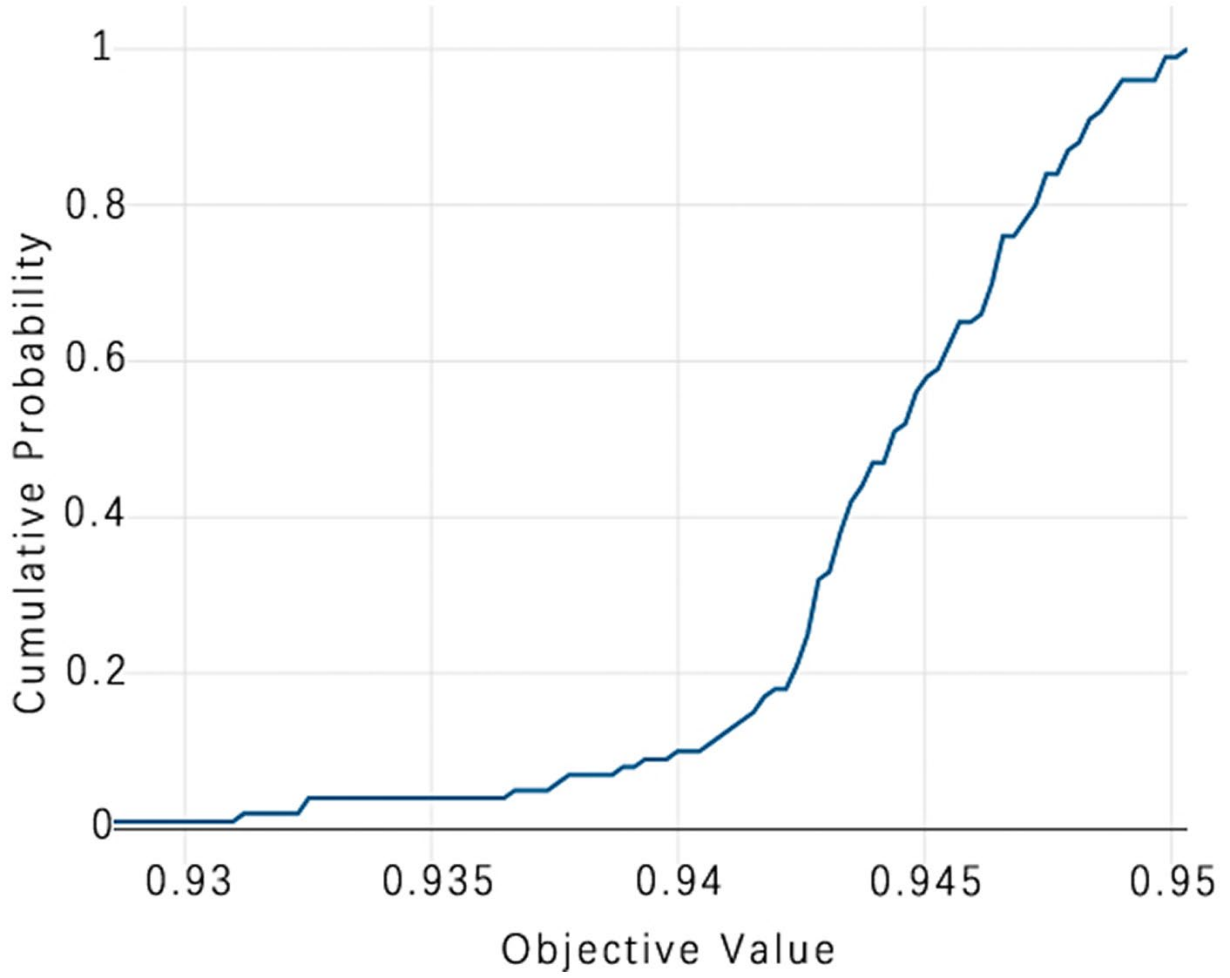
TPE_OptGBM model cumulative probability. (This chart is a Cumulative Distribution Function (CDF) graph that displays the cumulative probability of the objective function. As the cumulative probability approaches 1, the corresponding objective function value nears 0.95, indicating that almost all trials are able to achieve this value, which is used to assess the stability of model performance).

**Figure 13:**
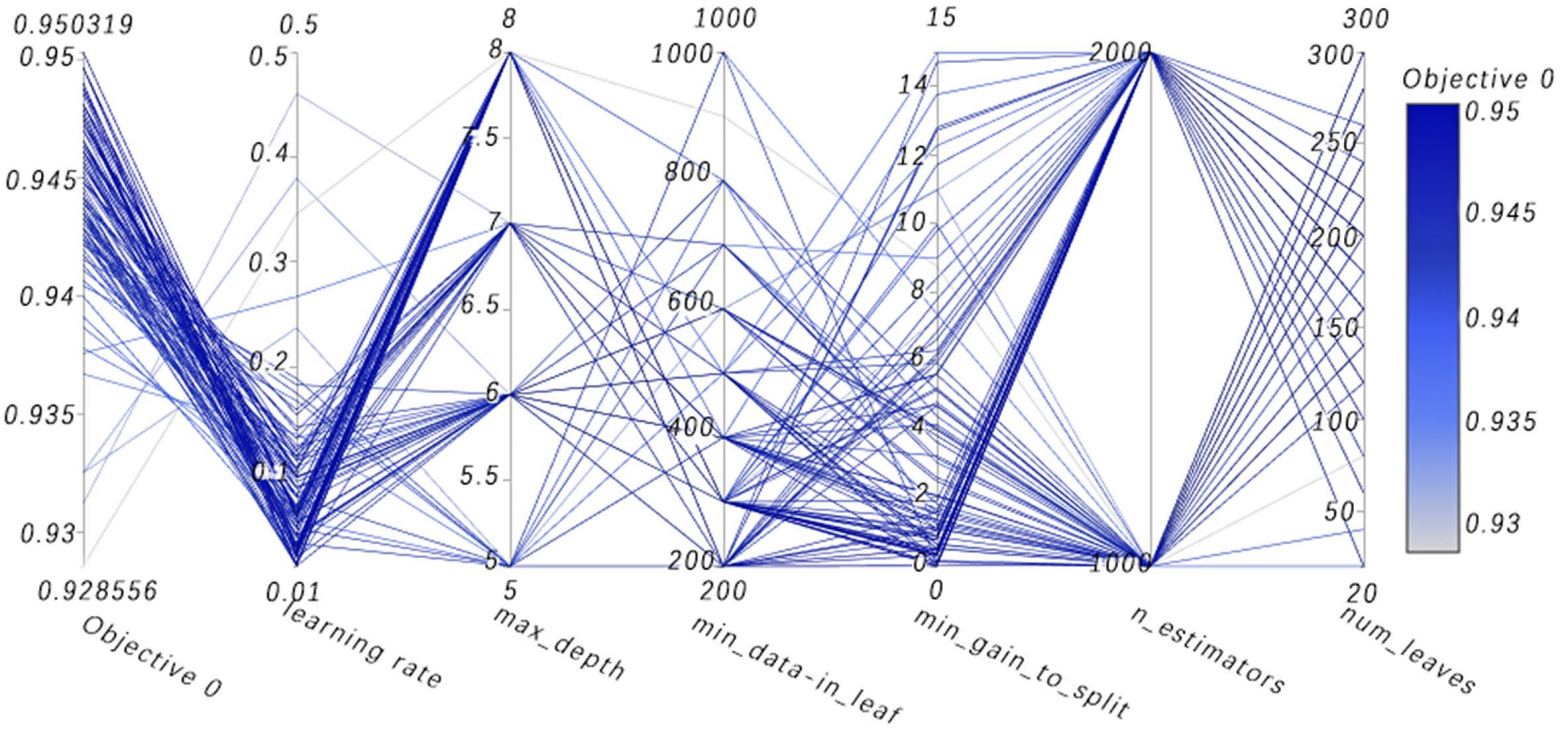
High-dimensional data visualizes parallel coordinates. (Visualization analysis of multidimensional data, where the path of the line shows how a set of parameters are combined together and their collective impact on the objective function value. By observing which combinations of parameters lead to higher or lower objective function values, one can gain insights into which parameter combinations are most critical for model performance).

The selection of model parameters has an important impact on model performance. In order to ensure that the model performs at an optimal level, a modified Bayesian hyperparameter optimization algorithm is used to find the optimal performance of the five key parameters of the TPE_OptGBM model. This step is crucial to ensure the performance and stability of the model. During the hyperparameter optimisation process, we randomly generated parameter values within the pre-given parameter range and performed 100 iterations to find the optimal parameter configurations. The results after parameter optimisation for different algorithms are shown in Table 2 (Figures 8–13).

**Table 2:** Parameters obtained by different algorithms.

Parametric	GridSampler	RandomSampler	TPESampler
n_estimators	1,000	1,000	2000
learning_rate	0.0421	0.3765	0.1748
max_depth	6	6	5
num_leaves	160	100	260
min_data_in_leaf	500	500	1,000
min_gate_to_split	8.7606	5.8583	0.1055

Figure 8 vividly demonstrates that, throughout the optimization process, as the number of trials increases, the performance of our model is enhanced to optimal levels through iterative refinement. The progression of finding the optimal value over time is distinctly visible. Figure 9 reveals the significant impact of learning rate variations on the model’s objective function value, underscoring the importance of adjusting the learning rate in optimizing model performance. By analyzing this trend, we can pinpoint the optimal learning rate, a pivotal step in the model tuning process. Figure 10, by depicting the temporal increase in the number of trials, allows us to observe periods of intensified experimentation, possibly indicating further exploration of specific parameter settings or an acceleration of the optimization process. Figure 11 highlights the substantial influence of the hyperparameters min_gain_to_split and learning_rate on model performance, emphasizing their critical roles in enhancing performance. The Cumulative Distribution Function (CDF) chart (Figure 12) offers a global perspective for assessing the stability of model performance, indicating that the majority of trials achieve or surpass the established performance threshold. Finally, Figure 13, through the visualization of multiple parameter combinations and their effects on the objective function value, enables us to identify the most crucial parameter combinations, which is vital for a deeper understanding of the model’s internal workings and for guiding future optimizations.

### 4.4 Comparative experiments

To ascertain the effectiveness of the model proposed in this study, we implemented a scientific and systematic approach for the selection of comparative models. This involved a detailed search through major scientific databases, followed by the randomized selection of five diverse models for comparative analysis. In recent years, these models have been widely applied in the field of sleep apnea detection research, ensuring the comprehensiveness and relevance of our comparison to this area of study, including Random Forest (Razi et al., 2021), LightGBM (Han and Oh, 2023), XGBoost (Yan et al., 2022), ResNet (Yang et al., 2022), and Bi-LSTM (Anbalagan et al., 2023). Using a comparison with these classical methods and deep learning models allows us to evaluate the performance of the new models and their advantages in problem solving. These benchmark methods, such as Random Forest, have been widely used in related fields, so using them as comparisons can reveal the advantages of the new model proposed in this paper for practical tasks. Meanwhile, the comparison with the deep learning model ResNet and Bi-LSTM can demonstrate the performance under different model classes, thus assessing their applicability more comprehensively. In addition, the comparison with models such as XGBoost can explore the potential advantages of different models on different types of problems. To ensure impartiality in the evaluation process, the proposed enhancement algorithm was integrated into each model under comparison and the best performance achieved by these comparative models was selected as the final outcome. The results are as follows (Table 3).

**Table 3:** Comparison of different models.

Modeling	ACC	Pre	Rec	F1
Random forest	85.9%	91.75%	75.50%	84.36%
LightGBM	87.82%	91.83%	84.87%	87.76%
Bi-lstm	89.71%	92.50%	90.36%	89.36%
ResNet	90.30%	91.90%	87.60%	89.70%
XGboost	93.51%	95.02%	92.02%	93.53%
**TPE_OptGBM**	**95.08%**	**94.80%**	**97.51%**	**96.14%**

The bold values represents the method we have proposed, and it is also the method that works best.

From the experimental results, it can be seen that our proposed TPE_OptGBM model performs well in several performance indicators. In particular, the TPE_OptGBM model achieves 95.08% in accuracy, 97.51% in recall, and 96.14% in F1 score. This means that our model is able to identify the patient’s condition with very high precision and accuracy in the sleep apnea detection task. Relative to other models, our model possesses higher accuracy and precision, which indicating that it is better at distinguishing between normal and abnormal samples during identification.

### 4.5 Ablation experiments

In order to verify the effectiveness of each module of the fusion model, we conducted ablation experiments to test the model performance by adding different algorithmic modules to the base model, step by step, to determine the actual effectiveness of the fusion model.

**Figure 14:**
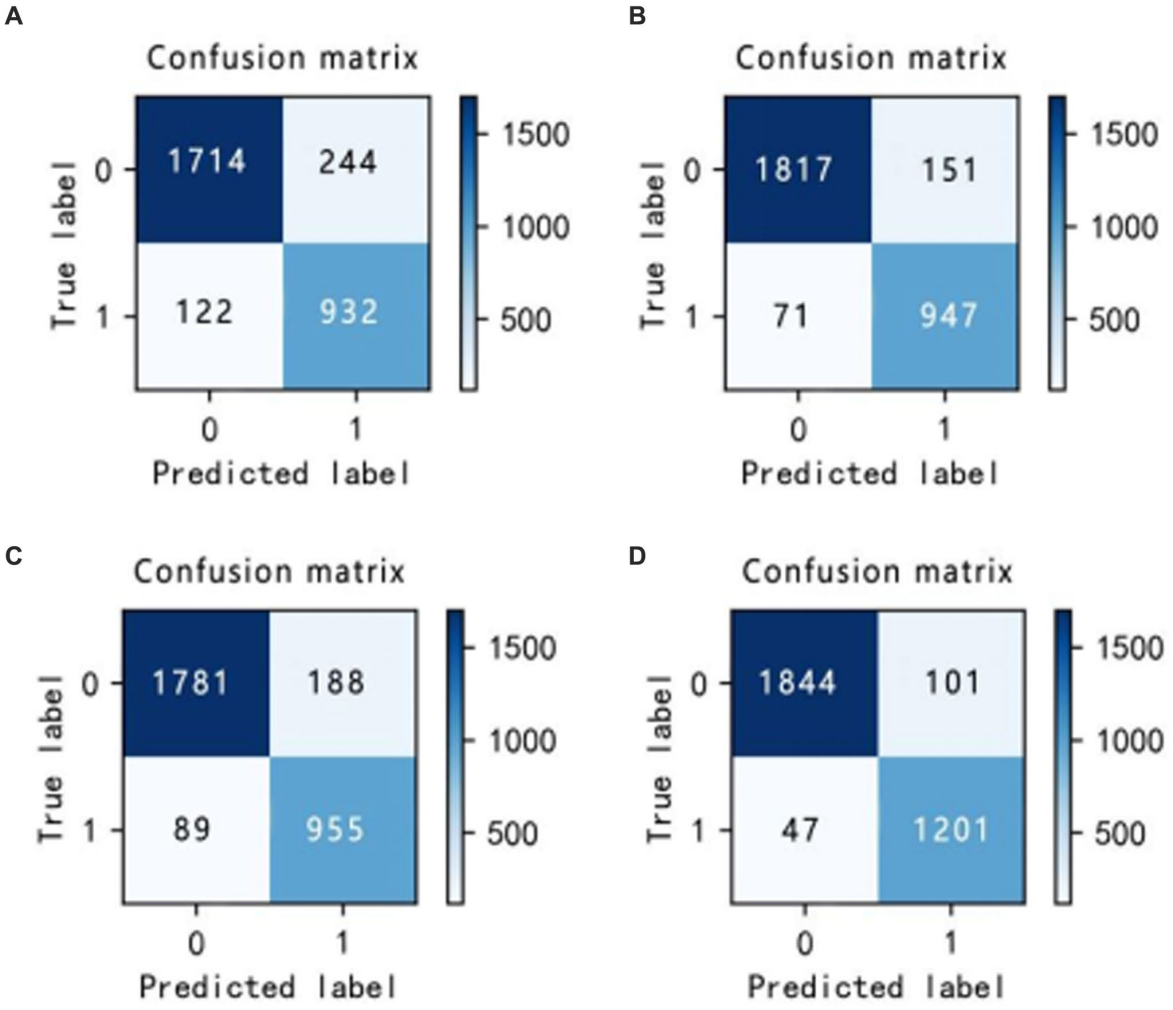
Confusion matrix incorporating different algorithms **(A–D)**.

**Figure 15:**
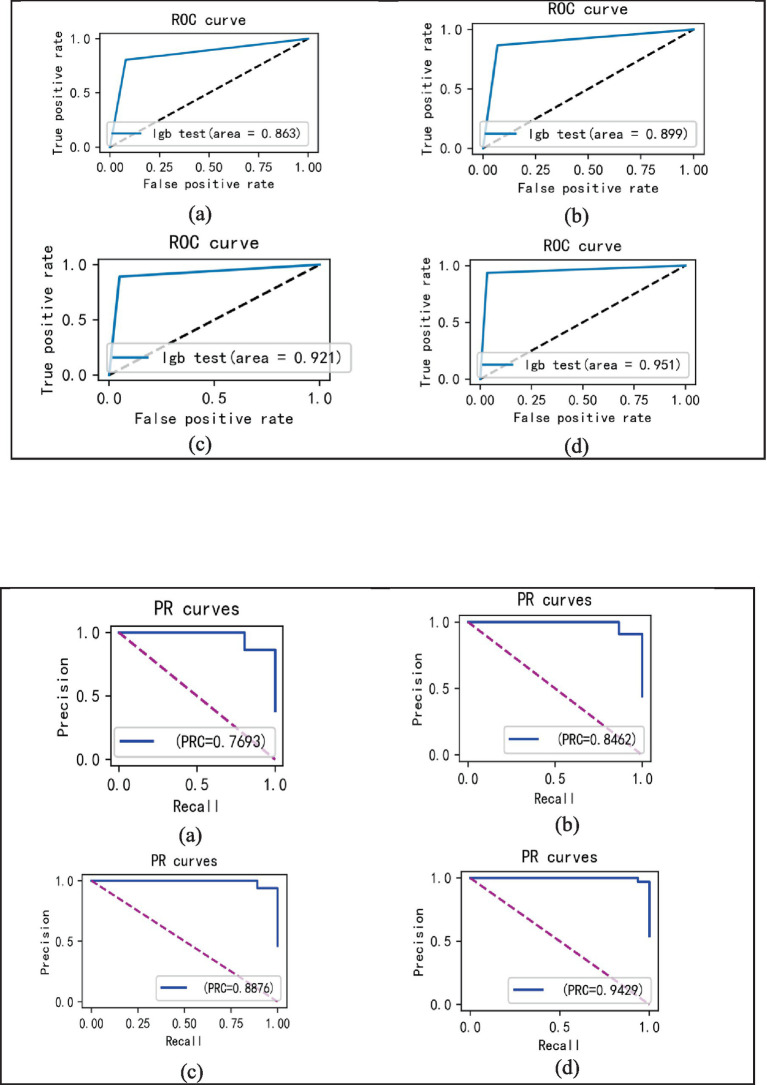
ROC curves incorporating different algorithms **(A–D)**.

**Figure 16:**
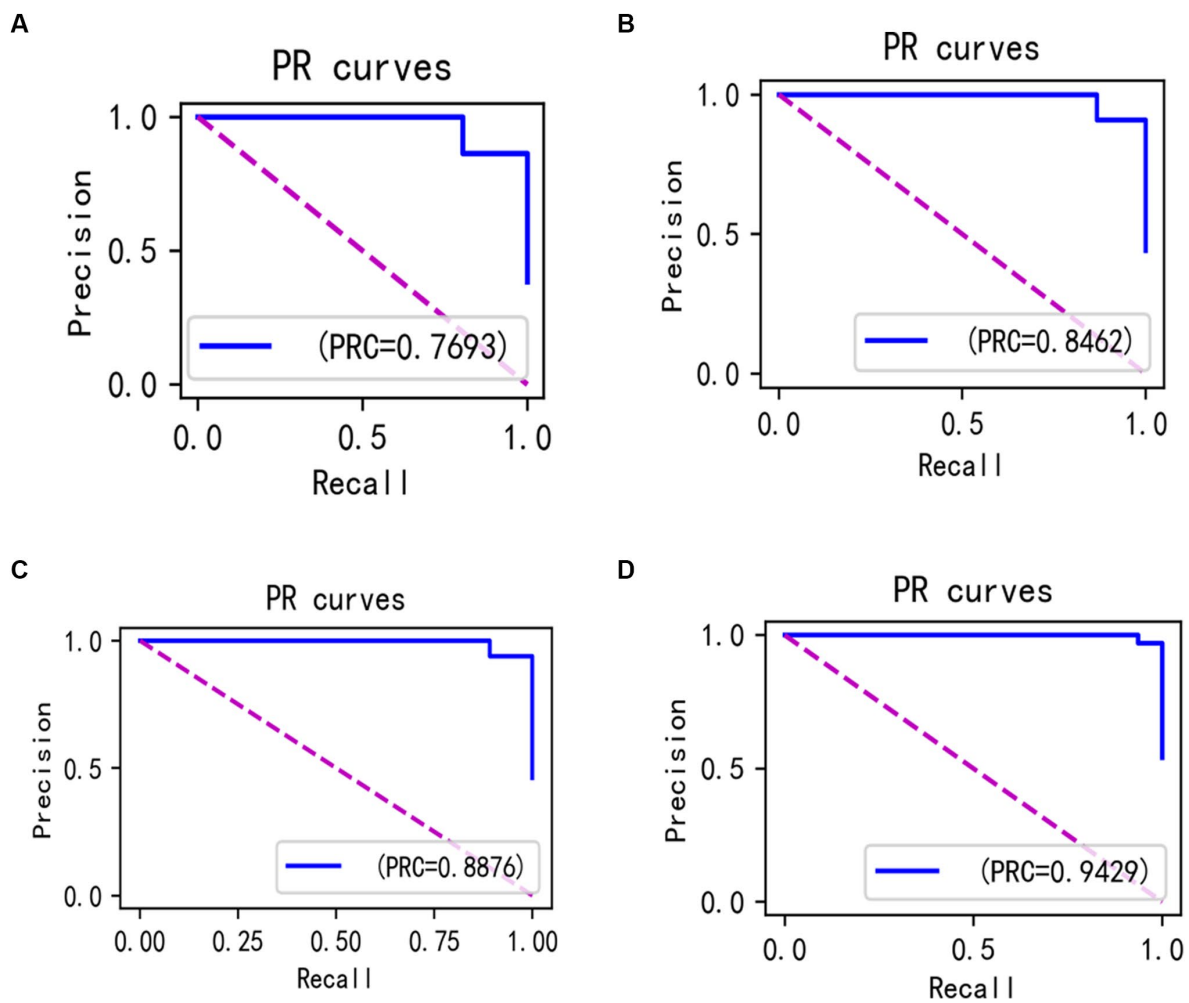
PR curves incorporating different algorithms **(A–D)**.

In the experiments, the Isolation Forest algorithm and the improved Bayesian hyperparameter optimization algorithm are introduced in this paper, and these algorithm modules are fused with the LightGBM model to improve the accuracy of the LightGBM model. Specifically, we separately fused the Isolation Forest algorithm and the Bayesian hyperparameter optimisation algorithm in our experiments, and then compared the fused model with the original and analyze changes before and after the introduction of algorithms. The comparison results are shown in Table 4 (Figures 14–16).

**Table 4:** Comparison of models with different modules.

Modeling	ACC	Pre	Rec	F1
LightGBM	87.82%	87.40%	93.35%	90.27%
A1-LightGBM	92.62%	92.32%	96.23%	94.23%
A2-LightGBM	90.83%	90.45%	95.24%	92.79%
**A1-A2-LightGBM**	**95.08%**	**94.80%**	**97.51%**	**96.14%**

The bold values represents the method we have proposed, and it is also the method that works best.

Let these two improvement methods be A1 and A2 respectively:

A1: Isolated forest algorithm.

A2: Improved Bayesian Hyperparametric Optimisation Algorithm.

In the laboratory, we visualized the performance of four different improved algorithms through confusion matrices, ROC curves, and Precision-Recall (PR) curves (Figures 14–16). Figure (a) shows the performance of the standard LightGBM algorithm; Figure (b) represents the performance of the A1-LightGBM algorithm; Figure (c) corresponds to the A2-LightGBM algorithm, which is the second improvement to the standard LightGBM; finally, Figure (d) demonstrates the performance of the A1-A2-LightGBM algorithm, which is the result of combining the previous two improvements applied to LightGBM. Table 5 shows the sample sizes of the a-d model training set and the test set.

**Table 5:** Training and testing sets for different algorithms.

	Training set	Test set
a	27,108	3,012
b	26,874	2,986
c	27,117	3,013
d	28,737	3,193

The results of the ablation experiments demonstrate that by using only the LightGBM model, a good performance was obtained, providing an accuracy of 87.82% and an F1 value of 90.27%. Subsequently, different improved algorithmic modules were gradually introduced. When the Isolated Forest algorithm (A1-LightGBM) was utilized, the accuracy rate was significantly improved to 92.62%, and the precision rate, AUC and F1 value were also significantly improved. Similarly, when the improved Bayesian Hyperparameter Optimization algorithm (A2-LightGBM) was introduced, the model performance was also improved to a certain extent in all indicators.

When we introduced both improved algorithmic modules into the LightGBM model (A1-A2-LightGBM), significant performance gains were observed, with accuracy reaching 95.08%, precision at 94.80%, AUC of 0.951, F1 value of 96.14%, and a peak recall performance of 97.51%. This demonstrates the superior outcomes achievable by integrating the Isolated Forest algorithm and the Bayesian Hyperparameter Optimization algorithm into the LightGBM model.

### 4.6 TPE_OptGBM fusion model

In the paper, we validate the performance of the TPE_OptGBM fusion model on a variety of metrics, including accuracy, precision, recall, F1 score, AUC (area under the curve), and ROC curve. We trained the model using a training set and evaluated its performance with a test set. Figure 8 shows the training and testing history curves of the TPE_OptGBM fusion model on each detection segment. It is worth noting that the difference in values is not large in each test, which indicates that the model has a good generalization performance and is not prone to overfitting. Meanwhile, we plotted the ROC curves of different networks to evaluate the performance of the model under different thresholds. By looking at the ROC curves, we can determine the AUC (area under the curve), which is a common metric for evaluating the performance of a classifier. In our experiments, it is observed that the TPE_OptGBM fusion model achieves the highest AUC value of 0.951. This indicates that our model performs well under different thresholds and has high classification ability. In order to verify the performance of proposed method, before the data was fed into the TPE_OptGBM model, we concatenated the training and testing sets, removed a portion of anomalous data, and then conducted 10-fold cross-validation, with 10% of the data being utilized as the test set. Through this process, we obtained the average accuracy as the final result. This step ensures more credibility and stability in the performance evaluation of the model.

## 5 Discussion

Sleep apnea has attracted a lot of attention in recent years, especially for its association with serious health problems such as cardiovascular disease and its early detection can be extremely helpful in the treatment and rehabilitation of the disease. The aim of this study was to explore an efficient and accurate method to detect sleep apnea to enable earlier intervention and treatment of this disorder. Experiments have also demonstrated that our proposed TPE_OptGBM model exhibits excellent performance in this task, and its effectiveness is not only reflected in the various performance indicators but also in the overall robustness of the model and its efficiency.

In the experimental section of this paper, we compare our proposed model with the current state-of-the-art methods, including Random Forest, LightGBM, XGBoost, Bi-LSTM and ResNet, and the results show that our proposed model has excellent results and achieves the best performance in terms of accuracy, precision, and F1 value. The model achieves better results due to our fully consideration of the following key factors at the beginning of the model design: (1) Applicability of the model. This model demonstrates effective capability for ECG data processing. ECG data samples can be perceived as two clusters in a high-dimensional space that overlap; for most of the characteristics of the obvious positive samples and negative samples, the model has a better ability to classify, and the cause of the difficulty in improving the classification accuracy is the differentiation between the part of the samples that are crossed. We have found that the tree model shows excellent classification accuracy for this kind of samples as shown on the experiments. Therefore, for ECG signals and other physiology, we can consider machine learning for prediction, especially the tree model, which may have better results. (2) We have fully considered the impact of the data and abandoned the practice of extracting sleep apnea information only from heart rate variability; instead, we integrated and extracted features from various perspectives, including the RRI, RAMP, and EDR signals, which makes the data input to the model to contain more original features, and the model can obtain better classification results based on multiple features. (3) We introduced the Isolation Forest algorithm to cope with the problem of anomalous data points present in the ECG signal. These anomalous data points are often disturbed by noise and are difficult to classify accurately. By introducing this method, we can ensure that it is still possible to get better results in the case of complex data. Furthermore, within the sleep apnea data set, the quantity of positive samples significantly outnumbers the negative ones, resulting on a disproportionate sample distribution. For deep learning models, under the premise that the ratio of one sample is higher than that of another sample, the model is more willing to believe that the sample belongs to a larger number of samples, which is also an important reason why it is difficult for deep learning to improve the accuracy rate. However, with the introduction of the Isolation Forest algorithm, this problem is successfully solved, providing a robust solution to the data analysis and classification problem. (4) The improved Parzen algorithm optimizes the parameters of the LightGBM algorithm. There are many parameters in the LightGBM model, and there are interactions and constraints among them, so how to find the important parameters and their specific values directly affect the performance of the model. How to find the important parameters and their specific values directly affect the model performance, so finding the optimal parameters of the model is key to maximize model performance. The traditional way is mainly based on the experience of manually adjusting the parameters and the results exhibit randomness, this paper adopts the latest hyper-parameter optimisation framework by comparing a variety of adjustment algorithms. This approach identifies the model parameters best suited for this task, leading to significant improvements in model performance.

In the ablation experiments, we found that each module of our proposed fusion model plays a key role in improving the overall performance of the model. The effectiveness of each module in the proposed model is verified through the ablation experiments. Table 4 lists the evaluation results of the models with different modules: when only the isolated deep forest algorithm is introduced, the numerical relationship shows that the accuracy is improved by 4.8%, which indicates that the algorithm is able to balance the data effectively. When only the improved Bayesian hyper-parameter optimisation algorithm is introduced, the accuracy is improved by about 3%, which shows that the introduced optimisation algorithm is able to accurately find the best parameters to fit the model. When both the Isolation Forest algorithm and the improved Bayesian hyperparameter optimization algorithm are incorporated, the fusion model’s accuracy notably increases to 95.08%, surpassing other current models. In terms of precision, recall and F1, 94.80, 97.51 and 96.14%, respectively, are higher than other models. Figure 14 shows the confusion matrix of the four models, and our proposed model produced the best results with far fewer samples with wrong predictions than the other models. From this, it can be concluded that our proposed model performs best in the classification of sleep apnea detection, which also implies that our model is not just a simple fusion, but obtains a significant improvement in overall performance with the introduction of each algorithm. In addition to this, compared to deep learning models, the model proposed in this paper improves overall classification performance, occupies less memory, it is faster and more efficient to train and can obtain higher accuracy rates while consuming less computer resources and time.

In the comparison of prior knowledge, we find that the model maintains a relatively high level of accuracy even without using any prior information. This indicates that our model does not completely depend on prior information, i.e., it has weak *a priori* properties, and it also indicates that the proposed model has good robustness.

In summary, the exceptional performance of our proposed TPE_OptGBM fusion model in sleep apnea detection is inextricably linked to the organic integration of its multiple factors. The model not only demonstrates strong performance in our area of interest, but also has the potential to be extended for application in other areas. Through experiments, we have shown that our method not only enhances the diagnostic accuracy of sleep apnea but also possesses the stability and flexibility to make it suitable for various practical scenarios. These include: (1) Integration into medical devices for comprehensive detection; (2) Development of portable mobile devices for sleep apnea detection.

In the future, we will further optimize the method, improve the classification accuracy of the model, and try to develop a more powerful sleep apnea diagnostic model to better assist doctors in clinical diagnosis. Meanwhile, developing portable wearable devices based on the reliable, stable and efficient features of this model in conjunction with hospitals and sleep centers is also the next step. Certainly, this study has several limitations which are reflected on the following aspects: (1) The limited sample size could potentially affect the generalizability of our results. Constrained by the availability of data, our model was trained and tested on a relatively small sample set, which may not provide a comprehensive evaluation of the model’s performance. This issue could potentially be mitigated through data augmentation and the recruitment of a larger number of participants for the study. (2) The model in this study was validated solely on the Apnea-ECG database. Despite encouraging outcomes on this database, performance on other databases remains untested. Different databases may exhibit diverse data distributions. Testing the model across various datasets enables an evaluation of its performance under different environments and conditions, thereby ascertaining the model’s robustness and applicability. (3) We implemented a specific preprocessing protocol in our study. Although we aimed to minimize the impact of preprocessing on the data, different preprocessing approaches could lead to varying outcomes, an area that warrants further investigation. (4) The computational complexity of the model is relatively high, posing potential challenges for real-time monitoring applications. Future research should focus on algorithm optimization to reduce resource consumption in practical applications.

## 6 Conclusion

Sleep apnea requires early detection and diagnosis for effective treatment and recovery. In this study, the characteristics of sleep apnea ECG signals are thoroughly investigated. Subsequently, we proposed an innovative model, TPE_OptGBM, which is capable of conveniently detecting sleep apnea solely through patients’ ECG signals. Experiments demonstrate that the model performs more efficently in several key metrics, including 95.08% accuracy, 94.80% precision, 97.51% recall, and 96.14% F1 score. The robustness of the model is fully validated by tests on balanced and unbalanced data and by cross-validation.

The advantages of the model proposed in this paper are as follows: (1) Our proposed model is designed for the diagnosis of sleep apnea and achieves the highest accuracy rate, effectively assisting doctors in making accurate diagnoses. (2) The TPE_OptGBM model proposed based on the idea of model fusion can effectively solve the key problems existing in the field of sleep apnea detection. (3) Compared to the deep learning model, our model has a faster training speed, which can obtain higher accuracy while occupying fewer computer resources and can provide reliability and high efficiency in the clinical process.

## Data availability statement

Publicly available datasets were analyzed in this study. This data can be found here: Apnea-ECG Database v1.0.0 (physionet.org).

## Author contributions

XX: Conceptualization, Methodology, Project administration, Writing – review & editing. AW: Conceptualization, Formal Analysis, Methodology, Writing – original draft. JH: Project administration, Writing – review & editing. CW: Data curation, Writing – review & editing. RL: Investigation, Writing – review & editing. ZS: Formal Analysis, Writing – original draft. JiaZ: Software, Writing – original draft. JinZ: Writing – original draft.
